# 1,4-Bis[(1*H*-pyrazol-1-yl)meth­yl]benzene

**DOI:** 10.1107/S1600536811022537

**Published:** 2011-06-18

**Authors:** Gui-Ying Dong, Tong-Fei Liu, Cui-Huan Jiao, Xiao-Chen Deng, Xiao-Ge Shi

**Affiliations:** a College of Chemical Engineering, Hebei United University, Tangshan 063009, People’s Republic of China; b Qian’an College, Hebei United University, Tangshan 063009, People’s Republic of China

## Abstract

In the title compound, C_14_H_14_N_4_, the center of the phenyl­ene group is a crystallographic center of inversion. The compound is composed of three aromatic rings displaying a Z-like conformation. The dihedral angle between the pyrazole rings and the central phenyl ring is 83.84 (9)°.

## Related literature

For background and coordination compounds with related ligands, see: Chang *et al.* (1993 ▶); Hou *et al.* (2010 ▶); Liu *et al.* (2011 ▶). For the crystal structure of the title compound with two solvent water mol­ecules, see: Shi *et al.* (2009 ▶).
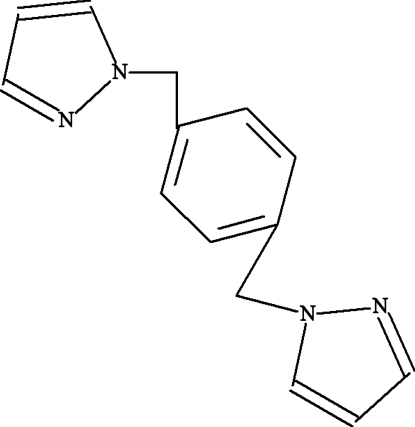


## Experimental

### 

#### Crystal data

C_14_H_14_N_4_
*M* *_r_* = 238.29
Monoclinic, 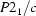

*a* = 5.6088 (8) Å
*b* = 6.8183 (10) Å
*c* = 16.526 (3) Å
β = 97.900 (15)°
*V* = 626.01 (17) Å^3^
*Z* = 2
Mo *K*α radiation
μ = 0.08 mm^−1^
*T* = 295 K
0.20 × 0.20 × 0.19 mm

#### Data collection

Bruker SMART CCD area-detector diffractometer
Absorption correction: multi-scan (*SADABS*; Sheldrick, 1996 ▶) *T*_min_ = 0.956, *T*_max_ = 0.996
2464 measured reflections
1109 independent reflections
580 reflections with *I* > 2σ(*I*)
*R*_int_ = 0.033

#### Refinement

*R*[*F* ^2^ > 2σ(*F* ^2^)] = 0.033
*wR*(*F* ^2^) = 0.064
*S* = 0.80
1109 reflections
83 parameters
1 restraint
H-atom parameters constrained
Δρ_max_ = 0.10 e Å^−3^
Δρ_min_ = −0.11 e Å^−3^

### 

Data collection: *SMART* (Bruker, 1998 ▶); cell refinement: *SAINT* (Bruker, 1998 ▶); data reduction: *SAINT*; program(s) used to solve structure: *SHELXS97* (Sheldrick, 2008 ▶); program(s) used to refine structure: *SHELXL97* (Sheldrick, 2008 ▶); molecular graphics: *SHELXTL* (Sheldrick, 2008 ▶); software used to prepare material for publication: *SHELXTL*.

## Supplementary Material

Crystal structure: contains datablock(s) I, global. DOI: 10.1107/S1600536811022537/im2296sup1.cif

Structure factors: contains datablock(s) I. DOI: 10.1107/S1600536811022537/im2296Isup2.hkl

Supplementary material file. DOI: 10.1107/S1600536811022537/im2296Isup3.cml

Additional supplementary materials:  crystallographic information; 3D view; checkCIF report

## Figures and Tables

**Fig. 1.**
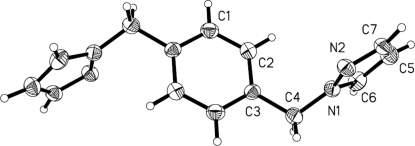
The molecular structure of (I), showing displacement ellipsoids at the 30% probability level for non-hydrogen atoms.

## supplementary crystallographic information

### Comment

Over the past few years, efforts have been focused on the investigation of coordination polymers with flexible ligands. Flexbile ligands with two or more pyrazolyl moieties such as 1,4-bis[(1*H*-pyrazol-1-yl)-methyl]-benzene find numerous applications in constructing metal–organic coordination polymers (Chang *et al.* 1993; Hou *et al.* 2010; Liu *et al.* 2011). The crystal structure of the title compound including two water molecules of hydration has previously been described (Shi *et al.* 2009). We report here the crystal structure of the title compound without any solvent molecules in the crystal lattice.

In (I), the center of the phenylene group is an inversion centre, so that the asymmetric unit consists of one-half of the title compound (Fig. 1). 1,4-Bis[(1*H*-pyrazol-1-yl)-methyl]-benzene is composed of three aromatic rings, displaying a Z shape, with the pyrazole rings on opposite sides of the plane of the phenyl ring. The whole molecule is nonplanar, the dihedral angle of the pyrazoles with respect to the central phenyl group are 83.84 (9)°. The average bond distances and angles for the pyrazole ring are in agreement with those of previously reported related pyrazole complexes and the hydrated title compound (Liu *et al.*2011; Shi *et al.* 2009). In contrast to the structure of the same compound with additional water molecules of hydration (Shi *et al.* 2009), the dihedral angles of the pyrazole units with respect to the central phenyl group are 76.9 (1)° and 74.5 (1)°, respectively. The hydrated compound further forms a two-dimensional supramolecular network by the hydrogen bond interactions including the water molecules.

### Experimental

(I) was obtained as an unexpected product in an attempt to construct a supramolecular Zn complex under hydrothermal conditions. A mixture of Zn(NO_3_)_2_.6H_2_O (166 mg, 1 mmol), phthalic acid (150 mg, 1 mmol), NaOH (80 mg,2 mmol) and 1,4-Bis[(1*H*-pyrazol-1-yl)-methyl]-benzene (238 mg, 1 mmol) in H_2_O (12 ml) was placed in a Teflon-lined stainless vessel and heated to 453 K for 72 h. Then, the reaction system was cooled to room temperature during 24 h to give rise to colourless crystals, which were collected and washed with water. Yield 0.024 g (10% of used (I)). Analysis calculated for C_14_H_14_N_4_ (238.29): C 70.57, H 5.92, N 23.51%; found: C 70.38, H 5.78, N 23.38%.

### Refinement

H atoms were placed in calculated positions, with C—H = 0.93 Å or C—H = 0.97 Å and refined with a riding model, with *U*_iso_(H) = 1.2*U*_eq_(C). Restraints (DELU) were applied to the *U_ij_* parameters of atoms C3 and C4.

### Crystal data

**Table:**

C_14_H_14_N_4_	*F*(000) = 252
*M**_r_* = 238.29	*D*_x_ = 1.264 Mg m^−^^3^
Monoclinic, *P*2_1_/*c*	Mo *K*α radiation, λ = 0.71073 Å
Hall symbol: -P 2ybc	Cell parameters from 1865 reflections
*a* = 5.6088 (8) Å	θ = 5.3–24.1°
*b* = 6.8183 (10) Å	µ = 0.08 mm^−^^1^
*c* = 16.526 (3) Å	*T* = 295 K
β = 97.900 (15)°	Block, colourless
*V* = 626.01 (17) Å^3^	0.20 × 0.20 × 0.19 mm
*Z* = 2	

### Data collection

**Table:**

Bruker SMART CCD area-detector diffractometer	1109 independent reflections
Radiation source: fine–focus sealed tube	580 reflections with *I* > 2σ(*I*)
graphite	*R*_int_ = 0.033
φ and ω scans	θ_max_ = 25.0°, θ_min_ = 3.2°
Absorption correction: multi-scan (*SADABS*; Sheldrick, 1996)	*h* = −6→6
*T*_min_ = 0.956, *T*_max_ = 0.996	*k* = −6→8
2464 measured reflections	*l* = −19→19

### Refinement

**Table:**

Refinement on *F*^2^	Secondary atom site location: difference Fourier map
Least-squares matrix: full	Hydrogen site location: inferred from neighbouring sites
*R*[*F*^2^ > 2σ(*F*^2^)] = 0.033	H-atom parameters constrained
*wR*(*F*^2^) = 0.064	*w* = 1/[σ^2^(*F*_o_^2^) + (0.0265*P*)^2^] where *P* = (*F*_o_^2^ + 2*F*_c_^2^)/3
*S* = 0.80	(Δ/σ)_max_ < 0.001
1109 reflections	Δρ_max_ = 0.10 e Å^−^^3^
83 parameters	Δρ_min_ = −0.11 e Å^−^^3^
1 restraint	Extinction correction: *SHELXL97* (Sheldrick, 2008), Fc^*^=kFc[1+0.001xFc^2^λ^3^/sin(2θ)]^-1/4^
Primary atom site location: structure-invariant direct methods	Extinction coefficient: 0.043 (3)

### Special details

**Table:**

Geometry. All e.s.d.'s (except the e.s.d. in the dihedral angle between two l.s. planes) are estimated using the full covariance matrix. The cell e.s.d.'s are taken into account individually in the estimation of e.s.d.'s in distances, angles and torsion angles; correlations between e.s.d.'s in cell parameters are only used when they are defined by crystal symmetry. An approximate (isotropic) treatment of cell e.s.d.'s is used for estimating e.s.d.'s involving l.s. planes.
Refinement. Refinement of *F*^2^ against ALL reflections. The weighted *R*-factor *wR* and goodness of fit *S* are based on *F*^2^, conventional *R*-factors *R* are based on *F*, with *F* set to zero for negative *F*^2^. The threshold expression of *F*^2^ > σ(*F*^2^) is used only for calculating *R*-factors(gt) *etc*. and is not relevant to the choice of reflections for refinement. *R*-factors based on *F*^2^ are statistically about twice as large as those based on *F*, and *R*-factors based on ALL data will be even larger.

### Fractional atomic coordinates and isotropic or equivalent isotropic displacement parameters (Å^2^)

**Table:**

	*x*	*y*	*z*	*U*_iso_*/*U*_eq_	
N2	0.3304 (2)	0.6599 (2)	0.19489 (8)	0.0484 (4)	
N1	0.1491 (2)	0.76019 (19)	0.15110 (8)	0.0450 (4)	
C2	0.3728 (3)	0.8298 (3)	0.00568 (11)	0.0536 (5)	
H2B	0.2880	0.7135	0.0088	0.064*	
C7	0.2330 (3)	0.4880 (3)	0.20879 (10)	0.0502 (5)	
H7A	0.3157	0.3862	0.2378	0.060*	
C1	0.5185 (3)	0.8528 (2)	−0.05491 (11)	0.0525 (5)	
H1A	0.5294	0.7517	−0.0920	0.063*	
C3	0.3522 (3)	0.9770 (3)	0.06120 (10)	0.0436 (4)	
C6	−0.0531 (3)	0.6553 (3)	0.13893 (11)	0.0555 (5)	
H6A	−0.1998	0.6959	0.1108	0.067*	
C4	0.1899 (3)	0.9606 (2)	0.12664 (11)	0.0562 (5)	
H4A	0.2606	1.0340	0.1742	0.067*	
H4B	0.0360	1.0203	0.1068	0.067*	
C5	−0.0058 (3)	0.4783 (3)	0.17507 (12)	0.0588 (5)	
H5A	−0.1111	0.3735	0.1767	0.071*	

### Atomic displacement parameters (Å^2^)

**Table:**

	*U*^11^	*U*^22^	*U*^33^	*U*^12^	*U*^13^	*U*^23^
N2	0.0428 (8)	0.0432 (10)	0.0573 (10)	0.0048 (8)	0.0001 (7)	0.0053 (8)
N1	0.0420 (8)	0.0448 (9)	0.0484 (9)	0.0056 (8)	0.0074 (7)	0.0057 (8)
C2	0.0598 (11)	0.0468 (12)	0.0558 (12)	−0.0024 (9)	0.0134 (10)	0.0014 (11)
C7	0.0593 (13)	0.0408 (12)	0.0504 (12)	0.0055 (10)	0.0072 (10)	0.0036 (10)
C1	0.0626 (11)	0.0460 (12)	0.0490 (12)	0.0036 (10)	0.0076 (10)	−0.0051 (10)
C3	0.0454 (10)	0.0443 (11)	0.0413 (11)	0.0087 (9)	0.0067 (8)	0.0027 (10)
C6	0.0352 (10)	0.0744 (15)	0.0559 (12)	0.0007 (11)	0.0025 (8)	−0.0021 (12)
C4	0.0635 (11)	0.0458 (12)	0.0614 (13)	0.0138 (9)	0.0165 (10)	0.0119 (10)
C5	0.0551 (13)	0.0566 (14)	0.0653 (13)	−0.0144 (10)	0.0100 (11)	−0.0042 (12)

### Geometric parameters (Å, °)

**Table:**

N2—C7	1.3264 (19)	C1—C3^i^	1.380 (2)
N2—N1	1.3504 (16)	C1—H1A	0.9300
N1—C6	1.3324 (18)	C3—C1^i^	1.380 (2)
N1—C4	1.4518 (19)	C3—C4	1.511 (2)
C2—C3	1.375 (2)	C6—C5	1.356 (2)
C2—C1	1.386 (2)	C6—H6A	0.9300
C2—H2B	0.9300	C4—H4A	0.9700
C7—C5	1.380 (2)	C4—H4B	0.9700
C7—H7A	0.9300	C5—H5A	0.9300
			
C7—N2—N1	104.04 (12)	C2—C3—C4	122.52 (16)
N2—N1—C6	111.81 (13)	C1^i^—C3—C4	119.39 (16)
N2—N1—C4	119.33 (14)	N1—C6—C5	107.47 (15)
C6—N1—C4	128.81 (16)	N1—C6—H6A	126.3
C3—C2—C1	120.76 (16)	C5—C6—H6A	126.3
C3—C2—H2B	119.6	N1—C4—C3	113.74 (14)
C1—C2—H2B	119.6	N1—C4—H4A	108.8
N2—C7—C5	111.86 (16)	C3—C4—H4A	108.8
N2—C7—H7A	124.1	N1—C4—H4B	108.8
C5—C7—H7A	124.1	C3—C4—H4B	108.8
C3^i^—C1—C2	121.18 (16)	H4A—C4—H4B	107.7
C3^i^—C1—H1A	119.4	C6—C5—C7	104.82 (16)
C2—C1—H1A	119.4	C6—C5—H5A	127.6
C2—C3—C1^i^	118.07 (15)	C7—C5—H5A	127.6

Symmetry codes: (i) −*x*+1, −*y*+2, −*z*.
